# Rewiring the bone marrow: Evolution and the transcriptional architecture of trained immunity

**DOI:** 10.7554/eLife.107551

**Published:** 2025-10-24

**Authors:** Sarah J Sun, Raúl Aguirre-Gamboa, Luis B Barreiro

**Affiliations:** 1 Committee on Immunology, University of Chicago Chicago United States; 2 Medical Scientist Training Program, University of Chicago Chicago United States; 3 Section of Genetic Medicine, Department of Medicine, University of Chicago Chicago United States; 4 Department of Human Genetics, University of Chicago Chicago United States; 5 Committee on Genetics, Genomics, and Systems Biology, University of Chicago Chicago United States; 6 Chan Zuckerberg Biohub Chicago Investigator Chicago United States; Murdoch Children's Research Institute Australia; National Institute of Immunology India

**Keywords:** trained immunity, epigenetics, transcription factors

## Abstract

The epigenetic adaptation of innate immune cells to inflammatory stimuli, or trained immunity, represents an evolutionarily conserved feature of host defense. Recent advances have revealed that such adaptations can occur at the level of hematopoietic stem and progenitor cells, resulting in long-lasting epigenetic reprogramming of the immune system. However, a comprehensive mechanistic understanding of these processes remains incomplete, limiting our capacity to predict or therapeutically manipulate the adaptive capacity of hematopoiesis. In this review, we survey the current literature to support a model of hematopoietic memory whose stimulus-specific nuances are shaped by specific cytokine environments and driven by the combinatorial activity of key transcription factors. Comparative analyses underscore the evolutionary conservation and essential biological roles of these factors, suggesting that trained immunity may reflect the strategic repurposing of ancient transcriptional programs for the purpose of enhancing host defense.

## Introduction

The innate immune system is the oldest branch of the broader human immune system and the sole source of immunity in most living species, including plants and invertebrates (Pancer and Cooper, 2006). In humans, quintessential innate immune cells, such as macrophages and dendritic cells (DCs), detect and ultimately eliminate pathogens via the expression of fixed, germline-encoded pathogen recognition receptors (PRRs), which confer innate immune cells with the ability to broadly recognize and phagocytose pathogenic microorganisms while preserving self-tolerance. Adaptive immune cells, such as T-cells, are more complex. They recombine germline-encoded sequences to generate an essentially infinite number of unique receptors with exquisite specificity. T-cells also exist in different states, such as ‘naïve’, ‘effector’, and ‘memory’, and the activation of a naïve cell by its specific antigen induces the permanent differentiation of the T-cell clone into a memory state via the induction of complex transcriptional, metabolic, and epigenetic remodeling.

Traditionally, this capacity for durable, antigen-specific memory was considered exclusive to the adaptive immune system. However, the field has evolved to appreciate the spectrum-like nature of memory (when loosely defined as short or long-term changes in immune cells that result from transient stimuli). All immune cells exhibit some ability to adapt, but the duration and specificity of these adaptations vary. Certain innate-like lymphocytes—including invariant natural killer T cells, mucosal-associated invariant T cells, and γδ T-cells—exhibit memory-like features despite utilizing a limited range of antigen receptors (Pellicci et al., 2020), blurring the classical boundaries between innate and adaptive immunity and highlighting the role of multiple types of memory in the immune response.

The expanding view of immune memory stems largely from discoveries made in the late 2000s and early 2010s, which revealed that classical innate immune cells can undergo short-term epigenetic, metabolic, and transcriptional reprogramming—typically lasting around 7 days—following PRR stimulation (Cheng et al., 2014; Foster et al., 2007; Quintin et al., 2012). These changes, in some cases, led to heightened inflammatory responses upon subsequent, nonspecific challenges (Cheng et al., 2014; Quintin et al., 2012). The term ‘trained immunity’ was coined in 2011 (Netea et al., 2011) to describe this phenomenon, which had long been observed in plants and invertebrates (Gourbal et al., 2018; Netea et al., 2019). Organisms as primitive as bacteria exhibit memory-like immunity through the CRISPR-Cas system, which incorporates viral DNA fragments into their genome to ‘remember’ and target future infections (Gourbal et al., 2018). In plants, a system called systemic acquired resistance enables adaptation to pathogens through epigenetic and transcriptional mechanisms (Gourbal et al., 2018). Evidence of innate immune memory has also been reported in organisms, such as mosquitoes and copepods, both of which rely exclusively on innate immunity (Gourbal et al., 2018; Netea et al., 2019). That such innate memory also existed within vertebrates was subsequently more formally demonstrated in human monocytes stimulated ex vivo with β-glucan (Cheng et al., 2014; Quintin et al., 2012) and shown to have an epigenetic and metabolic basis. These findings supported a broader plasticity across the immune system and, thus, a more complex evolutionary model in which the capacity for epigenetic adaptation to environmental stimuli is a basic feature of all immune cells across species, while the evolution of antigen-specific receptors marks a key innovation of adaptive immunity in vertebrates (Gourbal et al., 2018).

While in vitro studies provided evidence that short-lived innate immune cells could acquire nonspecific memory, their limited lifespan raised questions about the durability of such responses. This led to the hypothesis that long-term innate immune memory must be encoded in long-lived cell populations, such as tissue-resident innate cells and/or hematopoietic stem and progenitor cells (HSPCs). In 2018, several pivotal studies confirmed this hypothesis, showing that HSPCs can undergo persistent epigenetic, metabolic, and transcriptional reprogramming in response to inflammatory stimuli. These rewired HSPCs are capable of harboring molecular signatures of memory long-term through their ability to self-renew and are thought to continuously give rise to monocytes and neutrophils with enhanced innate immune responsiveness (Christ et al., 2018; Kaufmann et al., 2018; Mitroulis et al., 2018). This introduced a new conceptual framework: short-term, peripheral trained immunity (within mature innate immune cells) vs. a more long-term hematopoietic reprogramming wherein molecular adaptations encoded in long-lived stem and progenitor cells can be passed on to their mature immune progeny. Such progeny, in turn, often appear to harbor the classical characteristics of peripherally trained cells (Netea et al., 2020) and are capable of establishing long-term residence within inflamed tissues, further contributing to the longevity of innate immune memory (Patel et al., 2021). Together with a growing body of evidence that long-lived, self-renewing yolk-sac-derived tissue resident macrophages can harbor epigenetic memory of infections (Patel et al., 2021), the influx of reprogrammed bone-marrow-derived macrophages into inflamed tissues serves to fundamentally rewire innate immune responses within those tissues. This phenomenon is sometimes referred to as ‘central trained immunity’, although in this review, we will use the looser term of *hematopoietic reprogramming* to broaden our discussion to studies that, in our view, do not meet strict criteria for central trained immunity (see below for a more extensive discussion about terminology).

Extensive reviews have covered the triggers and mechanisms underlying peripheral trained immunity (Bekkering et al., 2018; Chavakis et al., 2022; Divangahi et al., 2021; Netea et al., 2020; Netea et al., 2016; Vuscan et al., 2024), as well as the growing body of work surrounding central trained immunity (Chavakis et al., 2022; Chavakis et al., 2019; Divangahi et al., 2021). However, a unified framework capable of explaining and predicting hematopoietic reprogramming across different contexts remains elusive. In this review, we aim to contribute to this ongoing discussion by focusing on memory within the hematopoietic system. In the first section, we adopt a mechanistic perspective to explore how specific stimuli can durably rewire hematopoiesis, altering innate immune output and function—emphasizing the roles of cytokine signaling and the activation of pioneering transcription factors (TFs). In the second section, we take a broader evolutionary and comparative view, highlighting the deep conservation of transcriptional machinery across species. We also explore emerging evidence of species-specific differences in hematopoietic memory mechanisms, particularly between mice and humans, and consider how these insights might guide future research in the field.

## Terminology: hematopoietic reprogramming

The term ‘(peripheral) trained immunity’, since first being coined in 2011, has classically been used to refer to the ability of a primary stimulus to directly reprogram innate immune cells to mount *stronger* cytokine responses to diverse secondary stimuli as compared to their naïve counterparts (Netea et al., 2011). This definition highlights the difference between trained immunity and the related phenomenon of innate immune tolerance in which strong lipopolysaccharide (LPS) stimulation leads to a temporary state of innate immune hypo*-*responsiveness to secondary stimulation (Foster et al., 2007; Novakovic et al., 2016). Another requirement is that cells return to a transcriptional baseline in between primary and secondary stimuli, retaining only epigenetic signatures imprinted by the primary stimulus to keep the chromatin in a more accessible, ‘ready’, state (Divangahi et al., 2021). Failure to return to a transcriptional baseline in between the primary and secondary stimulus has in some of the literature been termed ‘priming’ to distinguish it from the more strict definition of ‘training’ (Mishra and Ivashkiv, 2024).

Defining trained immunity of the hematopoietic system has proven to be more complex. While stimulus-induced changes within HSPCs do often lead to the production of innate immune cells which appear classically ‘trained’, the HSPCs themselves, from which these innate cells are derived, often retain residual changes in gene expression, TF activity, cell abundances, and lineage biases, even long after presumed clearance of the initiating stimulus. Relying on in vivo systems, it then also becomes difficult to determine whether residual inflammation from the primary stimulus may exist, or whether the residual changes in HSPCs represent a true, independent, rewiring of their transcriptional circuitry. Not surprisingly, existing studies have often used diverse indices of what it means for the bone marrow to be ‘trained’. Although the studies and examples discussed in this review have been categorized as examples of central trained immunity, we will from here on use the broader term *hematopoietic reprogramming* to acknowledge the fact that not all studies meet the definition of trained immunity in its strictest sense. *Hematopoietic reprogramming* (as a substitute for *central trained immunity*) will be used here to refer to long-lasting changes (this may include any combination of epigenetic, transcriptional, metabolic, or other persistent alterations) in HSPCs persisting in the absence of detectable levels of the primary stimulus, and leading to enhanced downstream innate immune cell function, composition, or responses to secondary stimuli.

## Cytokine-induced EM is integral to the establishment of hematopoietic reprogramming

Emergency myelopoiesis (EM) is believed to be an essential component of hematopoietic reprogramming. In general terms, it is defined as a reactive homeostatic response of HSPCs to increased myeloid cell demand (Manz and Boettcher, 2014). EM is therefore a central feature of systemic bacterial infections (Manz and Boettcher, 2014), and viral infections, such as COVID-19 (Schulte-Schrepping et al., 2020), which induce widespread inflammation and the depletion of neutrophils as they infiltrate into tissues. Noninfectious, iatrogenic causes such as myeloablative chemotherapy can also act as triggers of HSC proliferation and myeloid cell regeneration (Pietras et al., 2016), demonstrating that the mere act of myeloid cell depletion in the absence of infection is sufficient to drive this response. Mechanistically, peripheral signals of myeloid cell demand cause usually dormant, pluripotent long-term HSCs (LT-HSCs) to expand and give rise to myeloid-biased multipotent progenitors (MPPs). EM also involves the entry of normally quiescent lineage-committed granulocyte-monocyte precursors (GMPs) into the cell cycle (Swann et al., 2024). These events are largely thought to be coordinated by the action of key cytokines, including G-CSF, GM-CSF, M-CSF, IL6, IL-1β, TNFα, TGFβ, and type I and type II interferons (Manz and Boettcher, 2014; Park et al., 2024; Pascutti et al., 2016; Swann et al., 2024), although other cytokines, including IL4, have also been shown to coordinate EM under certain circumstances (LaMarche et al., 2024). Signaling by cytokines, either in combination or in isolation, results in cell proliferation at multiple points in the hematopoietic hierarchy, as well as the activation of lineage-determining TFs such as PU.1 (Heinz et al., 2010) and CEBPβ (Hirai et al., 2006), which bias stem cells toward the myeloid lineage (de Laval et al., 2020; Pietras et al., 2016; Yamashita and Passegué, 2019).

We believe that these processes, usually perceived as transient changes enabling the temporary mobilization of monocytes and granulocytes in times of need, are essential in the induction of persistent hematopoietic reprogramming and that cytokine-induced EM serves as a necessary, but likely not sufficient, trigger of hematopoietic reprogramming. In support of this hypothesis, our review of the literature (see examples below) has consistently demonstrated that an initial EM response precedes hematopoietic reprogramming. Most stimuli lead to the upregulation of at least one identifiable cytokine among IL1β, IFNγ, or IL6 (Chavakis et al., 2022; Chavakis et al., 2019; Zhao et al., 2014)—one possible exception being LPS which may directly act on HSCs via Toll-like receptor signaling (Chavakis et al., 2019; de Laval et al., 2020). Even so, TLR ligation is believed to lead to the production of cytokines such as IL6, which feedback to partially instruct EM via paracrine signaling (Chavakis et al., 2019; Zhao et al., 2014). In general, we find that differences in hematopoietic reprogramming between stimuli can be, to some extent, predicted by the cytokines they produce (Table 1).

**Table 1.** Cytokine-induced hematopoietic reprogramming. The table includes stimuli of hematopoietic reprogramming from studies for which cytokines were directly investigated. The phenotype-defining cytokine is bolded. Rows are colored according to cytokine (red—IL1β, yellow—IL6 ± IFN, blue—IFN in the absence of IL6). Stimuli that induce similar cytokine repertoires also produce similar reprogramming effects.

Stimulus	Species studied	Type	Key cytokines induced	Persistent reprogramming outcome	References
β-Glucan	Mice	Infectious mimic (PRR agonist) and inflammasome activator	**IL-1β,** G-CSF	Inherent LT-HSC myeloid bias; trained EM response to chemotherapy	Mitroulis et al., 2018
LIP	Mice	Sterile inflammation	**IL-1β**, G-CSF	Inherent LT-HSC myeloid bias; trained EM response to LPS; primed monocyte and neutrophil TNFα/IL-6 secretion upon LPS challenge	Li et al., 2022
BCG vaccination	Mice	Live attenuated infection	**IFN-γ**	Increased macrophage antimicrobial activity; IFN-γ dependent	Kaufmann et al., 2018
COVID-19 infection	Human	Viral infection	**IL-6**	Lasting monocyte chromatin remodeling and priming; persistent GMP expansion; reversed by IL-6 blockade	Cheong et al., 2023
Western diet	Mice	Metabolic trigger	**IL-6**, **IFN-γ, IL-1β,** G-CSF, GM-CSF	Lasting monocyte priming; GMP chromatin remodeling, differential gene expression, monocyte skewing	Christ et al., 2018

β-Glucan and ligature-induced periodontitis (LIP), for example, are seemingly unrelated mouse models of hematopoietic reprogramming. Both, however, induce IL1β and similar hematopoietic reprogramming phenotypes. β-Glucan and LIP both induce a persistent myeloid bias at the level of LT-HSCs that is transplantable into naïve recipients and prime the hematopoietic system to more rapidly increase myeloid cell production in response to secondary challenges with either chemotherapy or LPS. Blockage of IL1β signaling abrogates the reprogramming of long-lived myeloid bias in response to β-glucan (Mitroulis et al., 2018) and in the model of LIP also reduces the primed secretion of TNFα and IL6 by LIP-bone marrow-derived neutrophils and monocytes in response to LPS (Li et al., 2022). In further support of the essential role of IL1β in encoding these responses at the level of LT-HSCs, studies have shown that IL1β itself is sufficient to drive the expression of PU.1, as well as macrophage colony-stimulating factor (M-CSF) and granulocyte-macrophage colony-stimulating factor (GM-CSF) within long-term hematopoietic stem cells (LT-HSCs) to drive an intrinsic myeloid bias (Pietras et al., 2016) at the very apex of the hematopoietic hierarchy.

Other stimulus-cytokine pairs further support the notion that the action of cytokines dictates hematopoietic reprogramming. In contrast to IL1β, inducers of IFNγ and IL6 appear to generate a hematopoietic reprogramming phenotype that more predominantly involves monocyte- (rather than neutrophil) skewed myeloid cell production and GMP/monocyte-centric reprogramming.

For example, vaccination with *Bacillus* Calmette-Guérin (BCG), a live attenuated version of *Mycobacterium bovis,* induces hematopoietic reprogramming in a manner that is dependent on IFNγ (Kaufmann et al., 2018). In line with IFNγ’s intrinsic ability to polarize the bone marrow toward monocyte over granulocyte production, the main hematopoietic reprogramming phenotype of BCG is the production of macrophages with an increased ability to resist *Mycobacterium tuberculosis* infection in vitro even in mice treated with antibiotics, an effect which is lost completely in IFNγR KO mice (Kaufmann et al., 2018).

COVID-19 induces hematopoietic reprogramming via a mechanism that appears to involve IL6. Often acting in concert with IFNγ, IL6 acts directly on MPPs by downregulating production of the TFs Cebpa and Runx1, thus further skewing downstream GMPs toward the monocyte lineage (de Bruin et al., 2012). Accordingly, COVID infection can induce the persistence of elevated GMPs detected in the peripheral circulation for at least 4–12 months following exposure and leads to the production of epigenetically modified monocytes primed to secrete elevated levels of IL6 and TNF-α in response to TLR7/8 agonist R848 or IFNα (Cheong et al., 2023). Treatment with Tocilizumab—an antibody which blocks IL6 signaling—leads to significantly reduced levels of the GMPs. Moreover, in a mouse model of COVID infection, concomitant treatment with anti-IL6 significantly decreased the post-COVID burden of inflammatory myeloid cells within both the lungs and brain (Cheong et al., 2023).

Western diet is an interesting example of a stimulus that induces multiple cytokines simultaneously, including IL6, IFNγ, and IL1β. In line with the tendency for IL6 and IFNγ to reprogram HSPCs at the level of the GMP and monocyte, western diet hematopoietic reprogramming is also characterized by lasting effects on the chromatin accessibility and gene profiles of GMPs isolated even 4 weeks after return to a control diet, GMP skewing toward the monocyte lineage, and priming of both monocytes and GMPs. Although IL1β was markedly increased in this study, whether its effects on LT-HSCs resembled those of other IL1β producers, such as β-glucan, remains unclear (Christ et al., 2018).

Altogether, the data suggest that the specific flavor of hematopoietic reprogramming that is induced by any given stimulus is influenced by the cytokines which they induce (Table 1). β-Glucan and LIP converge on IL-1β and in both examples, result in LT-HSCs with persistent myeloid bias and primed EM response to secondary challenges (Li et al., 2022; Mitroulis et al., 2018). Mycobacteria, which induce the monocyte/macrophage-polarizing cytokine IFNγ when injected intravenously, train the bone marrow of mice to give rise to macrophages with increased bacterial killing capacity (Kaufmann et al., 2018). Models of human COVID infection and western diet in mice implicate IL-6 as a key cytokine in reprogramming the bone marrow at the level of monocytes and GMPs (Cheong et al., 2023; Christ et al., 2018). When taken together, existing data suggests that blocking the signaling of key cytokines involved in programming demand-adapted myeloid cell mobilization abrogates both EM and the ability of the hematopoietic system to become reprogrammed, suggesting that EM may be a necessary component of hematopoietic reprogramming. In some cases, administration of recombinant cytokines, such as IFNγ (Kain et al., 2023), has also reproduced phenotypes of hematopoietic reprogramming, thereby also suggesting sufficiency. Nonetheless, there remains a need for more systematic necessity and sufficiency experiments to further delineate the exact role of each EM-inducing cytokine in hematopoietic reprogramming.

## Remodeling hematopoiesis: how TFs encode innate immune memory

### The pioneer TF machinery of EM drives hematopoietic reprogramming

In addition to cytokine signaling, the induction of myeloid lineage directing TFs is an essential step in hematopoietic reprogramming. These include TFs such as PU.1 and C/EBPβ, which are notable for their ability to act as *pioneer factors* by binding to previously inaccessible chromatin and setting off a cascade of subsequent TF binding events. These and other pioneer factors are believed to act in concert to remodel the chromatin landscape such that the regulatory enhancers and promoters of myeloid cells remain open, while those of lymphoid lineage cells become inaccessible. Studies suggest that the same network of pioneering TFs may simultaneously imprint the physical chromatin ‘scars’ that underlie the phenotypes of hematopoietic reprogramming (Larsen et al., 2021). In taking a systematic look at the TFs implicated in hematopoietic reprogramming across studies, we find that they are almost exclusively limited to pioneer TFs with previously known roles as, or acting in concert with, lineage-determining TFs (used to denote a subtype of pioneer factor which dictates hematopoietic lineage fate).

In an attempt to gain clarity, we have organized these TFs into four groups according to the specificity with which they are activated:

1. C/EBPβ, which is a lineage-determining TF specific to and required for EM.
2. STATs, which are critical in cytokine-induced EM but also important in mounting inflammatory responses in many other contexts outside of hematopoiesis.
3. The AP-1 TFs FOS and JUN, which are pioneer factors ubiquitously induced not only in EM but in response to most stimuli.
4. Universally cooperative factors—SP/KLF, EGR, MAZ, and ZNF—which are not specifically implicated in EM but have been shown to universally engage in highly cooperative binding and in prolonging the chromatin binding time of their partner TFs (Table 2).

**Table 2.** Transcription factors (TFs) implicated in hematopoietic reprogramming fall into four groups. These range from TFs that are activated and bind chromatin in specific contexts (such as emergency myelopoiesis [EM] for CEBPB) to those that engage in widespread binding and play roles in dictating the global chromatin landscape (stripe factors). Check marks on the right indicate TF involvement within each study.

TF group	TF function specificity	Key members	Role in hematopoietic reprogramming	Models/Stimuli with evidence of TF group involvement					
				Mouse				Human	
				β-Glucan	BCG	LPS	Western diet	BCG	COVID
Lineage	+	C/EBP (β, α, ε, δ)	Required for cytokine-induced EM; downstream of G-CSF through STAT3	✓		✓		✓	✓
Cytokine responsive	+ +	STAT1, STAT3, STAT5	Mediates cytokine signaling (IL6, IFNs, G-CSF)		✓		✓	✓	✓
Ubiquitous pioneer	+ + +	FOS/JUN (AP-1)	Induced in many stimuli; drive enhancer formation and chromatin access			✓	✓	✓	✓
Universal stripe	+ + +	KLFs, SPs, EGRs, ZNFs	Enables through co-binding other TFs shaping epigenetic landscape				✓	✓	✓

To briefly elaborate on the functions of these four groups of TFs: The first group consists of C/EBPb—a member of the C/EBP family of TFs which share a highly conserved C-terminus leucine zipper dimerization domain, adjacent to a basic DNA binding domain (bZIP) (Landschulz et al., 1989). The most important role of C/EBPb is in cytokine-induced emergency granulopoiesis (Hirai et al., 2006). It is induced in response to G-CSF (universally important in EM and often induced concomitantly with other cytokines) signaling which triggers CEBPβ activation through the activation of JAK-STAT3. C/EBPb and STAT3 bind to the promoters of various cell cycle genes, such as c-Myc, leading to GMP proliferation (Manz and Boettcher, 2014; Zhang et al., 2010). C/EBPb is also induced within HSPCs higher up the differentiation tree, such as in LT-HSCs (de Laval et al., 2020). C/EBPε has also been implicated in some studies and is involved in the terminal differentiation of granulocytes (Yamanaka et al., 1997).

STAT TFs differ from C/EBP in that they not only orchestrate EM, but also play a dual role in driving inflammatory gene expression responses in response to cytokine stimulation of terminally differentiated cells. JAK inhibition in the context of cytokine stimulation, which blocks the ability to activate STATs, prevents PU.1 binding and the deposition of histone modifications (Ostuni et al., 2013), providing further evidence that chromatin opening in response to inflammatory cytokines is instigated by STATs. In the context of EM, STATs 1, 3, and 5 are directly upregulated by G-CSF stimulation (Manz and Boettcher, 2014). IL6 also induces the expression of Stat3 (which binds to regulatory regions together with C/EBPβ as discussed above), and GM-CSF upregulates STAT5a and STAT5b. Interferons activate STAT1, which can form a complex with STAT2 and IRF9 (ISGF3), which recruits chromatin remodelers SWI/SNF, HATs, and HDACs (Philips et al., 2022).

The third group of TFs, AP-1 TFs, has established roles as pioneer factors with de novo enhancer formation in EM but is also generally responsive to most stimuli in most tissues, and thus modulates chromatin in a plethora of contexts. Thus, they can be differentiated from STATs in that they respond to a broader range of stimuli. For example, exposure of mouse fibroblasts to serum induces the AP-1 binding to several de novo sites and association of AP-1 with components of the BAF chromatin remodeling complex (Vierbuchen et al., 2017). Ap-1 TFs are often reported to bind in combination with more specific TFs. In a survey of other studies, it was noted that factors co-enriched with AP-1 are often cell-type specific, such as C/EBPb in macrophages and NFAT in T-cells (Vierbuchen et al., 2017), suggesting that AP-1 may instigate chromatin opening to invite a second layer of more specific TF binding.

The final group of TFs is connected by their universal cooperative binding patterns. A recent study (Zhao et al., 2022) called such factors ‘stripe’ factors for the prominent vertical lines they formed on colocalization heatmaps as a result of their tendency to colocalize with most other TFs. Notable members included KLF, SP, EGR, ZNF, ZBTB, MAZ, PATZ1, and RREB1—all containing C2H2 zinc finger domains. SNPs affecting universal stripe factor binding have been shown to have a larger impact on chromatin accessibility than did SNPs targeting other TFs (Zhao et al., 2022), suggesting an integral role of USFs in dictating global chromatin architecture.

### Stimulus-specific combinations of TFs shape hematopoietic reprogramming

The TF groups described above are not activated in isolation but are selectively engaged depending on the stimulus and the cytokines it induces. Below, we summarize evidence from key models of hematopoietic reprogramming to illustrate how these transcriptional programs are deployed in vivo.

The stimuli that lead to hematopoietic reprogramming engage various combinations of TFs from these classes. LPS in mice, for example, leads to persistent changes in chromatin accessibility in LT-HSCs that can be transplanted into naïve hosts to confer significant protection against *Pseudomonas aeruginosa* infection. These sites of altered chromatin accessibility enrich significantly for CEBPB, as well as the AP-1 members FOS and JUN (de Laval et al., 2020). The hematopoietic reprogramming stimulus β-glucan (which induces persistent LT-HSC bias in an IL1β-dependent manner as described above) induces changes in the gene expression of CEBPε targets after 7 days, again implicating the CEBP family (Mitroulis et al., 2018). In mice, BCG most predominantly modulates the expression of STAT1 downstream of IFNγ to reprogram bone marrow-derived macrophages to engage in heightened mycobacterium killing capacity (Kaufmann et al., 2018). Of note, epidermal skin stem cells form epigenetic memories in response to topical imiquimod via the formation of STAT3, FOS, and JUN complexes. Once activated by imiquimod, STAT3 recruits FOS and JUN, with JUN acting as a bookmarker. On heterologous stimulation, FOS rapidly reassociates with JUN, enabling faster gene expression to secondary injury (Larsen et al., 2021). While not demonstrated to the same level of mechanistic detail within hematopoietic stem cells, the example is consistent with the observation that small combinations of TFs drawn from STATs, AP-1, CEBP, or stripe factor TFs can orchestrate epigenetic memories.

Other stimuli engage broader swaths of TFs across larger number of classes. The universal stripe factors KLF4 and EGR1, concomitant with FOS/JUN and STAT TF activities, were implicated in western diet-reprogrammed GMPs after 4 weeks (Christ et al., 2018). Human studies, primarily of COVID or BCG, of hematopoietic reprogramming have also provided invaluable insights into the range of TFs implicated in hematopoietic reprogramming. COVID, for which IL6 signaling is critical for hematopoietic reprogramming, engages TFs of all four classes. Blockage of IL6 reduces the level of persistent C/EBPε and C/EBPβ activities found in late samples (4–12 months post-COVID), implicating the CEBP TFs downstream of IL6 in hematopoietic reprogramming. COVID infection also induces persistent changes in chromatin accessibility around STAT3, increased activity of FOS and JUN in HSPCs and monocytes 4–12 months post infection, and differential expression of KLF2 in HSPC 4–12 months post infection (Cheong et al., 2023).

Similarly, human intradermal vaccination with BCG leads to persistent changes in the chromatin accessibility of CMPs and GMPs at sites that enrich for TFs of all four classes (including CEBPB, STAT1, FOS, JUN, and TF motifs in the KLF/SP and EGR families) but for which stripe factors have the strongest enrichments. Out of the 11 TF motif families enriched within CMP and GMP progenitors with concomitant evidence of altered TF activity within HSCs upstream, six, including ‘ZNF/MAZ’, ‘KLF/SP_2’, ‘EGR1/2/3/4’, ‘KLF/SP_1’, ‘KLF2/3/6’, and ‘ZNF24’, overlapped those labeled as universal stripe factors (Sun et al., 2024). Thus, it appears that in the context of subcutaneous BCG vaccination of humans, persistently altered activity of stripe factors in HSCs and downstream changes in chromatin accessibility within progenitors at sites bound by these TFs is a key feature of the resultant hematopoiesis reprogramming.

Taken together, these studies suggest that all stimuli of hematopoietic reprogramming converge on the need to engage chromatin remodeling through pioneer or cooperative TFs. While this mechanism appears conserved, the specific TFs engaged differ by stimulus, likely due to variation in the cytokine milieu. In support of this hypothesis, both western diet and COVID-19 infections implicate IL-6 as upregulated cytokines, and both engage TFs from larger numbers of classes (3–4) compared to, for example, the IL1β inducer β-glucan, or the interferon inducer BCG in mice (Table 2). Still, the current body of literature emphasizes the need for larger numbers of studies which use the same stimuli in both mice and humans and which collect the same measurements (many studies measure cytokines or TFs but not both, and some measure TF activity through gene expression while others use chromatin accessibility measurements) to decrease the number of confounding variables that complicate interpretation of the current literature.

## Species may matter: TF usage in mouse vs. human hematopoietic reprogramming

**Figure 1.**
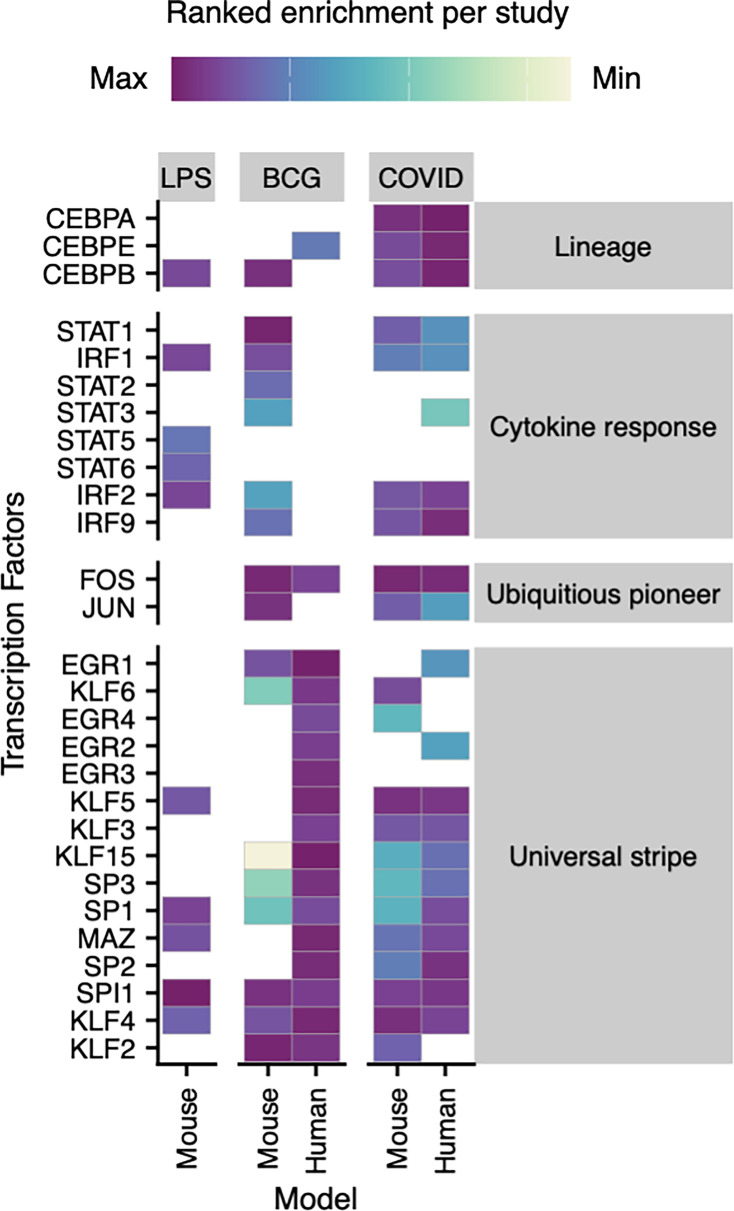
Heatmap of human and mouse studies with transcription factors (TFs) colored according to their ranked motif or expression (Bacillus Calmette–Guérin [BCG] mouse) enrichment within hematopoietic stem and progenitor cells (HSPCs) 1 or more months following training with the indicated stimulus. Each column represents a different study. Studies are grouped by stimulus type (top gray boxes). Species (mouse or human) is indicated on the bottom x-axis. White squares indicate no significant enrichment (NE).

While Table 2 suggests that hematopoietic reprogramming is driven by different combinations of TFs because of different stimuli, we also reasoned that species-specific factors, rather than specific stimuli, may explain some of these differences. We noticed that the studies in mice (Christ et al., 2018; de Laval et al., 2020; Kaufmann et al., 2018; Mitroulis et al., 2018) generally contained TF representatives from fewer numbers of our TF classes compared to the studies in humans (Cheong et al., 2023; Sun et al., 2024). These TF classes were, of course, artificially defined by us and not based on any formal structural or evolutionary similarity. In an attempt to more objectively compare TF usage in hematopoietic reprogramming across species, we analyzed TF motif enrichment data from three studies spanning human and mouse HSPCs, alongside TF differential expression data from HSCs in Kaufmann et al. (which did not include chromatin accessibility data) and a mouse dataset of COVID infection published alongside the human data in Cheong et al. Studies in Table 2 without published datasets on TF expression or motif enrichment were not included. For each of the five included datasets, TF motif enrichment FDR values—or differential gene expression FDR values in the case of Kaufmann et al.—were ranked from most to least significant. This ranking enabled cross-study comparisons of preferential TF usage. We then focused on a combined set of TFs implicated in the literature as important in hematopoietic reprogramming in either mouse, human, or both, as shown in Figure 1. Viewing the data in this manner, albeit limited by small sample sizes in differences in data modalities, revealed an overall consistent usage of TFs across species and conditions. Hierarchical clustering suggests that differences between studies are likely influenced not only by stimulus but also species (Figure 1), thus TF utilization in hematopoietic reprogramming may be shaped by a complex interplay of factors, including stimulus type (live vs. inert), stimulus class (virus vs. bacteria), stimulus dose, and host species (mouse vs. human). While some TFs were enriched in a study-specific manner (e.g. EGR3 being solely enriched within human HSPCs following BCG vaccination), others displayed enrichment patterns suggestive of specificity to one species or infectious stimulus. FOS was enriched within all studies involving live infectious agents, but not in LPS injection. TFs such as MAZ and SP1 were most strongly enriched in the human studies compared to mice, being among the top quarter of most enriched motifs in humans vaccinated with BCG or infected with COVID-19, but not their mouse counterparts. In support of the idea that human hematopoietic reprogramming may involve larger complexes of factors, human studies had larger numbers of total enriched TFs compared to mouse studies of the same stimulus (human/mouse: BCG = 746/635; COVID 423/378; LPS NA/97). Nonetheless, the highly congruent TF enrichments between mouse and human COVID-infected HSPCs (both published in Jeong et al.) suggest that a significant portion of species-specific differences may be confounded by differences in the stimuli typically used. Extended versions of such analyses could provide better context for understanding the roles of different enriched TFs as either species or stimulus-specific responders.

## Selective constraint on TFs involved in trained immunity

Despite some evidence for species-specific differences in TF usage in hematopoietic reprogramming, the data in Figure 1 suggest a high degree of overall similarity between humans and mice, especially when comparing studies that used the same stimulus (e.g. COVID). This functional conservation reinforces the biological relevance of the trained immunity program across species.

A major goal in human genetics is to identify genes that are important for disease susceptibility and fitness. One useful way to assess the biological importance of a gene is to measure its level of selective constraint—i.e., how strongly natural selection acts to eliminate deleterious variants from the population (Agarwal et al., 2023; Fuller et al., 2019). Genes that are under strong purifying selection will show a marked depletion of loss-of-function (LOF) variants, indicating that disruptions to their function reduce fitness and are thus selected against. We reasoned that if the genes central to trained immunity, such as the cytokines and TFs discussed above, are important for human immune adaptation and ultimately survival, particularly in the context of infectious disease, they should exhibit strong selective constraint. To test this, we analyzed gene-level LOF constraint metrics derived from the gnomAD v2.1 dataset, which includes exome sequences from approximately 125,000 individuals spanning 19,071 protein-coding genes (Karczewski et al., 2020). Specifically, we examined *shet*, a measure of the fitness reduction associated with carrying one copy of an LOF variant in each gene (Zeng et al., 2024). Larger *shet* values indicate stronger selection against LOF variants and, by extension, greater importance to fitness; smaller values suggest either tolerance to disruption or functional redundancy.

**Figure 2.**
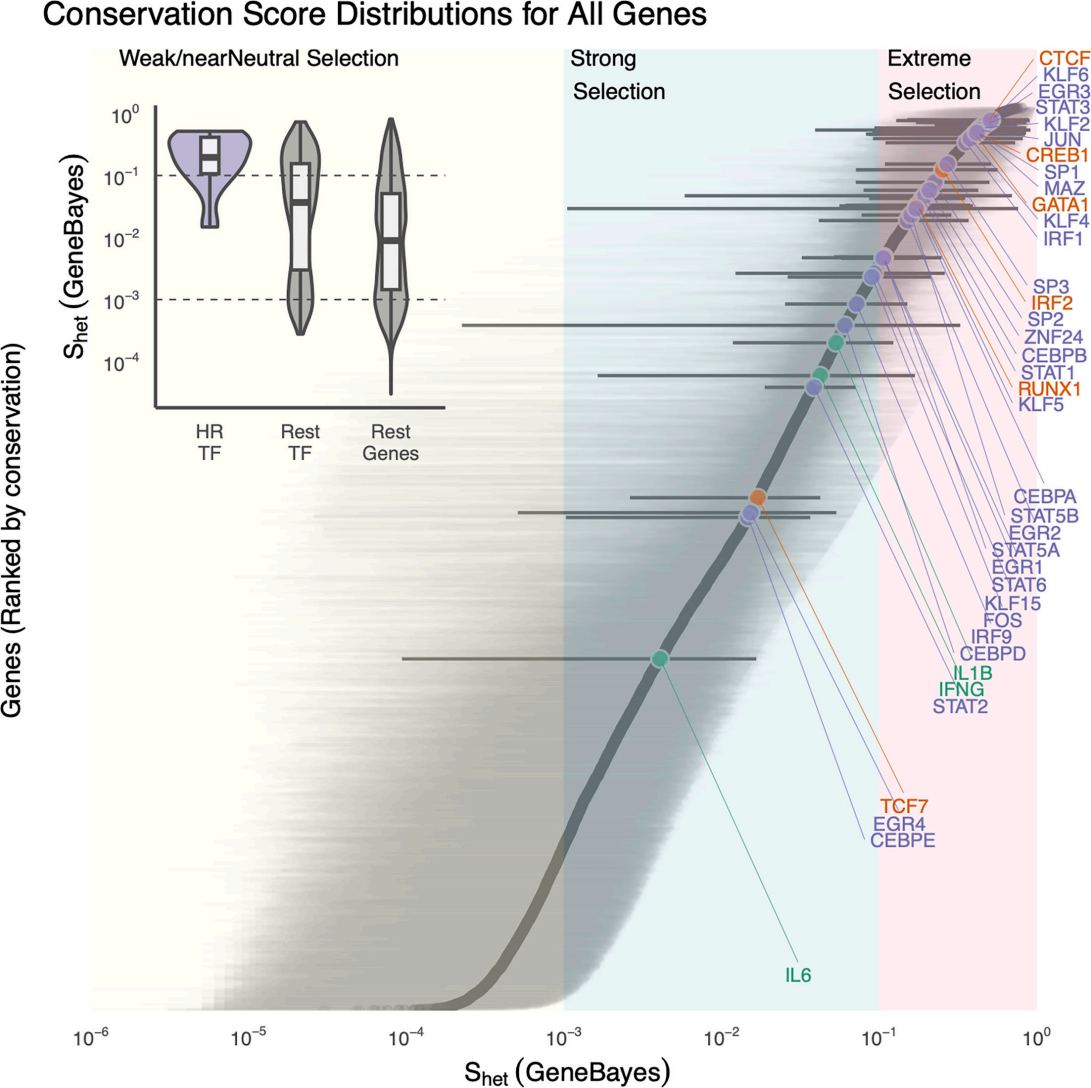
Conservation scores (*shet*) for cytokines (green) and transcription factors (TFs) (purple) implicated across mouse and human studies of hematopoietic reprogramming, compared to all other genes (gray). The figure shows posterior distributions of *shet* for individual genes, ordered by mean. Lines represent 95% credible intervals, with labeled genes represented by thick black lines. Colors represent the selection regimes. The inset panel summarizes *shet* values for TFs involved in hematopoietic reprogramming (HR-TFs), comparing them to all other TFs and to the rest of the genome.

We focused on the key effector molecules and TFs (described above in this review) as core components of the trained immunity program. When compared to the genome-wide distribution of *shet* values, we found that TFs involved in trained immunity show some of the highest levels of selective constraint across the human genome (Figure 2). Notably, this elevated constraint is not simply a reflection of their being TFs; even when compared specifically to all TFs in the genome, those implicated in trained immunity are significantly more constrained (Figure 2).

This suggests that these genes play essential roles in human fitness and survival. Stripe TFs such as KLF6, KLF2, EGR3, SP1, and MAZ are among the most constrained genes in the trained immunity network, and the genome overall.

These TFs are known to play critical and broadly pleiotropic roles in chromatin regulation, cell fate decisions, and stress responses, often in coordination with lineage-specific factors. For example, KLF6 is essential for hematopoietic stem cell quiescence and vascular development, and germline inactivation in mice results in embryonic lethality (Bialkowska et al., 2017). SP1 is a master regulator involved in the transcription of thousands of genes, including those required for immune responses, metabolism, and cell cycle control; mutations are associated with severe multisystem developmental syndromes. Finally, EGR3 regulates both neuronal development and T-cell activation; although rare, LOF mutations are linked to immune dysfunction and neurodevelopmental phenotypes (Li et al., 2007).

The extreme constraint on these genes underscores their nonredundant, dosage-sensitive roles, making even partial loss deleterious. While these TFs are involved in a range of biological processes beyond trained immunity, their central positioning in immune regulatory networks likely contributes to their evolutionary conservation. This supports the idea that mechanisms underpinning trained immunity—especially those related to chromatin remodeling and transcriptional reprogramminghave been subject to strong selective pressures, likely due to their impact on host survival in the face of infection.

In contrast, many effector molecules in the trained immunity network show lower levels of constraint, even though they play important roles in immune responses. For instance, IL6 and IL1β, both critical mediators of inflammatory responses and trained immunity, exhibit moderate to low *shet* values. This suggests that partial or even complete LOF variants in these genes are compatible with survival and may have relatively mild phenotypic consequences.

Indeed, common variants in IL6, IL1β, and related cytokine genes are found at appreciable frequencies in the general population, including missense and regulatory variants that modulate expression or function (Gong et al., 2022; Ołdakowska et al., 2022; Smith and Humphries, 2009). Some of these are associated with variation in infection susceptibility, inflammatory disease risk, or vaccine responsiveness, but they rarely cause severe monogenic disorders. For example, polymorphisms in the IL6 promoter have been associated with differences in CRP levels and outcomes in sepsis or COVID-19 but are not causative of immunodeficiency (Gong et al., 2022; Ołdakowska et al., 2022; Smith and Humphries, 2009).

This pattern is consistent with functional redundancy in cytokine networks: many cytokines share receptor subunits (e.g. IL-6 and IL-11 signaling through gp130) or activate overlapping downstream pathways such as JAK-STAT (Murakami et al., 2019). In this modular architecture, TFs act as essential convergence points, integrating signals from diverse upstream cues, while individual cytokines may be more expendable or context-specific.

Taken together, these data support a model in which trained immunity relies on a robust and evolutionarily constrained transcriptional core, built on TFs that are indispensable for orchestrating broad and durable responses. In contrast, upstream effector molecules exhibit greater flexibility, reflecting both their redundancy and the adaptive value of variation in cytokine responsiveness across diverse human environments.

## Conclusion and future directions

The extension of trained immunity to the hematopoietic compartment has revealed that diverse stimuli—both inert and infectious—can durably reprogram the lineage biases, epigenetic landscapes, and transcriptional circuits of LT-HSCs. While this diversity of stimuli has posed interpretive challenges, it has also clarified common mechanisms: hematopoietic reprogramming is consistently initiated by EM, typically coordinated by cytokines such as IL-1β, IL-6, and IFN-γ acting on LT-HSCs. Downstream, the activation of pioneer and lineage-determining TFs capable of remodeling chromatin likely constitutes the next critical step in encoding immune memory.

Comparative analyses suggest that mouse models often engage fewer TFs than human studies—whether this reflects true species-specific biology, experimental tractability, or differences in stimulus remains unclear. However, many of the TFs implicated in hematopoietic reprogramming, particularly those involved in chromatin remodeling and lineage specification, are among the most evolutionarily constrained genes in the human genome. This constraint likely reflects their indispensable roles in core developmental and cellular processes, rather than any selection pressure specific to trained immunity. Instead, trained immunity may have emerged, at least in part, as a byproduct of the reuse of this deeply conserved transcriptional architecture in the context of infection and inflammation.

Looking ahead, trained immunity offers a powerful framework for exploring fundamental questions: How is epigenetic memory maintained in self-renewing cells? How do cells preserve core identity while flexibly adapting to environmental challenges? Self-sustaining TF circuits—like the Myc-Wnt/β-catenin loop in embryonic stem cells (Fagnocchi et al., 2016) or the IRF-KLF-SPI network in DC development (Lin et al., 2015)—may provide a useful blueprint. Whether similar circuits exist in reprogrammed HSPCs remains to be explored.

A key frontier lies in decoding the thresholds that separate transient adaptation from long-term memory. Are these governed by the intensity, duration, or type of stimulusor by a combinatorial logic yet to be defined? Elucidating these parameters will not only advance our understanding of hematopoietic reprogramming and trained immunity, but also inform broader questions in immunology and regenerative medicine, from T-cell fate decisions to the design of durable cellular therapies and improved patient stratification strategies.

## References

[bib1] Agarwal I, Fuller ZL, Myers SR, Przeworski M (2023). Relating pathogenic loss-of-function mutations in humans to their evolutionary fitness costs. eLife.

[bib2] Bekkering S, Arts RJW, Novakovic B, Kourtzelis I, van der Heijden CDCC, Li Y, Popa CD, Ter Horst R, van Tuijl J, Netea-Maier RT, van de Veerdonk FL, Chavakis T, Joosten LAB, van der Meer JWM, Stunnenberg H, Riksen NP, Netea MG (2018). Metabolic induction of trained immunity through the mevalonate pathway. Cell.

[bib3] Bialkowska AB, Yang VW, Mallipattu SK (2017). Krüppel-like factors in mammalian stem cells and development. Development.

[bib4] Chavakis T, Mitroulis I, Hajishengallis G (2019). Hematopoietic progenitor cells as integrative hubs for adaptation to and fine-tuning of inflammation. Nature Immunology.

[bib5] Chavakis T, Wielockx B, Hajishengallis G (2022). Inflammatory modulation of hematopoiesis: linking trained immunity and clonal hematopoiesis with chronic disorders. Annual Review of Physiology.

[bib6] Cheng S-C, Quintin J, Cramer RA, Shepardson KM, Saeed S, Kumar V, Giamarellos-Bourboulis EJ, Martens JHA, Rao NA, Aghajanirefah A, Manjeri GR, Li Y, Ifrim DC, Arts RJW, van der Veer BMJW, Deen PMT, Logie C, O’Neill LA, Willems P, van de Veerdonk FL, van der Meer JWM, Ng A, Joosten LAB, Wijmenga C, Stunnenberg HG, Xavier RJ, Netea MG (2014). mTOR- and HIF-1α-mediated aerobic glycolysis as metabolic basis for trained immunity. Science.

[bib7] Cheong JG, Ravishankar A, Sharma S, Parkhurst CN, Grassmann SA, Wingert CK, Laurent P, Ma S, Paddock L, Miranda IC, Karakaslar EO, Nehar-Belaid D, Thibodeau A, Bale MJ, Kartha VK, Yee JK, Mays MY, Jiang C, Daman AW, Martinez de Paz A, Ahimovic D, Ramos V, Lercher A, Nielsen E, Alvarez-Mulett S, Zheng L, Earl A, Yallowitz A, Robbins L, LaFond E, Weidman KL, Racine-Brzostek S, Yang HS, Price DR, Leyre L, Rendeiro AF, Ravichandran H, Kim J, Borczuk AC, Rice CM, Jones RB, Schenck EJ, Kaner RJ, Chadburn A, Zhao Z, Pascual V, Elemento O, Schwartz RE, Buenrostro JD, Niec RE, Barrat FJ, Lief L, Sun JC, Ucar D, Josefowicz SZ (2023). Epigenetic memory of coronavirus infection in innate immune cells and their progenitors. Cell.

[bib8] Christ A, Günther P, Lauterbach MAR, Duewell P, Biswas D, Pelka K, Scholz CJ, Oosting M, Haendler K, Baßler K, Klee K, Schulte-Schrepping J, Ulas T, Moorlag Simone JCFM, Kumar V, Park MH, Joosten LAB, Groh LA, Riksen NP, Espevik T, Schlitzer A, Li Y, Fitzgerald ML, Netea MG, Schultze JL, Latz E (2018). Western diet triggers NLRP3-dependent innate immune reprogramming. Cell.

[bib9] de Bruin AM, Libregts SF, Valkhof M, Boon L, Touw IP, Nolte MA (2012). IFNγ induces monopoiesis and inhibits neutrophil development during inflammation. Blood.

[bib10] de Laval B, Maurizio J, Kandalla PK, Brisou G, Simonnet L, Huber C, Gimenez G, Matcovitch-Natan O, Reinhardt S, David E, Mildner A, Leutz A, Nadel B, Bordi C, Amit I, Sarrazin S, Sieweke MH (2020). C/EBPβ-dependent epigenetic memory induces trained immunity in hematopoietic stem cells. Cell Stem Cell.

[bib11] Divangahi M, Aaby P, Khader SA, Barreiro LB, Bekkering S, Chavakis T, van Crevel R, Curtis N, DiNardo AR, Dominguez-Andres J, Duivenvoorden R, Fanucchi S, Fayad Z, Fuchs E, Hamon M, Jeffrey KL, Khan N, Joosten LAB, Kaufmann E, Latz E, Matarese G, van der Meer JWM, Mhlanga M, Moorlag Simone JCFM, Mulder WJM, Naik S, Novakovic B, O’Neill L, Ochando J, Ozato K, Riksen NP, Sauerwein R, Sherwood ER, Schlitzer A, Schultze JL, Sieweke MH, Benn CS, Stunnenberg H, Sun J, van de Veerdonk FL, Weis S, Williams DL, Xavier R, Netea MG (2021). Trained immunity, tolerance, priming and differentiation: distinct immunological processes. Nature Immunology.

[bib12] Fagnocchi L, Cherubini A, Hatsuda H, Fasciani A, Mazzoleni S, Poli V, Berno V, Rossi RL, Reinbold R, Endele M, Schroeder T, Rocchigiani M, Szkarłat Ż, Oliviero S, Dalton S, Zippo A (2016). A myc-driven self-reinforcing regulatory network maintains mouse embryonic stem cell identity. Nature Communications.

[bib13] Foster SL, Hargreaves DC, Medzhitov R (2007). Gene-specific control of inflammation by TLR-induced chromatin modifications. Nature.

[bib14] Fuller ZL, Berg JJ, Mostafavi H, Sella G, Przeworski M (2019). Measuring intolerance to mutation in human genetics. Nature Genetics.

[bib15] Gong B, Huang L, He Y, Xie W, Yin Y, Shi Y, Xiao J, Zhong L, Zhang Y, Jiang Z, Hao F, Zhou Y, Li H, Jiang L, Yang X, Song X, Kang Y, Tuo L, Huang Y, Shuai P, Liu Y, Zheng F, Yang Z (2022). A genetic variant in IL-6 lowering its expression is protective for critical patients with COVID-19. Signal Transduction and Targeted Therapy.

[bib16] Gourbal B, Pinaud S, Beckers GJM, Van Der Meer JWM, Conrath U, Netea MG (2018). Innate immune memory: an evolutionary perspective. Immunological Reviews.

[bib17] Heinz S, Benner C, Spann N, Bertolino E, Lin YC, Laslo P, Cheng JX, Murre C, Singh H, Glass CK (2010). Simple combinations of lineage-determining transcription factors prime cis-regulatory elements required for macrophage and B cell identities. Molecular Cell.

[bib18] Hirai H, Zhang P, Dayaram T, Hetherington CJ, Mizuno S, Imanishi J, Akashi K, Tenen DG (2006). C/EBPbeta is required for “emergency” granulopoiesis. Nature Immunology.

[bib19] Kain BN, Tran BT, Luna PN, Cao R, Le DT, Florez MA, Maneix L, Toups JD, Morales-Mantilla DE, Koh S, Han H, Jaksik R, Huang Y, Catic A, Shaw CA, King KY (2023). Hematopoietic stem and progenitor cells confer cross-protective trained immunity in mouse models. iScience.

[bib20] Karczewski KJ, Francioli LC, Tiao G, Cummings BB, Alföldi J, Wang Q, Collins RL, Laricchia KM, Ganna A, Birnbaum DP, Gauthier LD, Brand H, Solomonson M, Watts NA, Rhodes D, Singer-Berk M, England EM, Seaby EG, Kosmicki JA, Walters RK, Tashman K, Farjoun Y, Banks E, Poterba T, Wang A, Seed C, Whiffin N, Chong JX, Samocha KE, Pierce-Hoffman E, Zappala Z, O’Donnell-Luria AH, Minikel EV, Weisburd B, Lek M, Ware JS, Vittal C, Armean IM, Bergelson L, Cibulskis K, Connolly KM, Covarrubias M, Donnelly S, Ferriera S, Gabriel S, Gentry J, Gupta N, Jeandet T, Kaplan D, Llanwarne C, Munshi R, Novod S, Petrillo N, Roazen D, Ruano-Rubio V, Saltzman A, Schleicher M, Soto J, Tibbetts K, Tolonen C, Wade G, Talkowski ME, Neale BM, Daly MJ, MacArthur DG, Genome Aggregation Database Consortium (2020). The mutational constraint spectrum quantified from variation in 141,456 humans. Nature.

[bib21] Kaufmann E, Sanz J, Dunn JL, Khan N, Mendonça LE, Pacis A, Tzelepis F, Pernet E, Dumaine A, Grenier JC, Mailhot-Léonard F, Ahmed E, Belle J, Besla R, Mazer B, King IL, Nijnik A, Robbins CS, Barreiro LB, Divangahi M (2018). BCG educates hematopoietic stem cells to generate protective innate immunity against tuberculosis. Cell.

[bib22] LaMarche NM, Hegde S, Park MD, Maier BB, Troncoso L, Le Berichel J, Hamon P, Belabed M, Mattiuz R, Hennequin C, Chin T, Reid AM, Reyes-Torres I, Nemeth E, Zhang R, Olson OC, Doroshow DB, Rohs NC, Gomez JE, Veluswamy R, Hall N, Venturini N, Ginhoux F, Liu Z, Buckup M, Figueiredo I, Roudko V, Miyake K, Karasuyama H, Gonzalez-Kozlova E, Gnjatic S, Passegué E, Kim-Schulze S, Brown BD, Hirsch FR, Kim BS, Marron TU, Merad M (2024). An IL-4 signalling axis in bone marrow drives pro-tumorigenic myelopoiesis. Nature.

[bib23] Landschulz WH, Johnson PF, McKnight SL (1989). The DNA binding domain of the rat liver nuclear protein C/EBP is bipartite. Science.

[bib24] Larsen SB, Cowley CJ, Sajjath SM, Barrows D, Yang Y, Carroll TS, Fuchs E (2021). Establishment, maintenance, and recall of inflammatory memory. Cell Stem Cell.

[bib25] Li L, Yun SH, Keblesh J, Trommer BL, Xiong H, Radulovic J, Tourtellotte WG (2007). Egr3, a synaptic activity regulated transcription factor that is essential for learning and memory. Molecular and Cellular Neurosciences.

[bib26] Li X, Wang H, Yu X, Saha G, Kalafati L, Ioannidis C, Mitroulis I, Netea MG, Chavakis T, Hajishengallis G (2022). Maladaptive innate immune training of myelopoiesis links inflammatory comorbidities. Cell.

[bib27] Lin Q, Chauvistré H, Costa IG, Gusmao EG, Mitzka S, Hänzelmann S, Baying B, Klisch T, Moriggl R, Hennuy B, Smeets H, Hoffmann K, Benes V, Seré K, Zenke M (2015). Epigenetic program and transcription factor circuitry of dendritic cell development. Nucleic Acids Research.

[bib28] Manz MG, Boettcher S (2014). Emergency granulopoiesis. Nature Reviews. Immunology.

[bib29] Mishra B, Ivashkiv LB (2024). Interferons and epigenetic mechanisms in training, priming and tolerance of monocytes and hematopoietic progenitors. Immunological Reviews.

[bib30] Mitroulis I, Ruppova K, Wang B, Chen LS, Grzybek M, Grinenko T, Eugster A, Troullinaki M, Palladini A, Kourtzelis I, Chatzigeorgiou A, Schlitzer A, Beyer M, Joosten LAB, Isermann B, Lesche M, Petzold A, Simons K, Henry I, Dahl A, Schultze JL, Wielockx B, Zamboni N, Mirtschink P, Coskun Ü, Hajishengallis G, Netea MG, Chavakis T (2018). Modulation of myelopoiesis progenitors is an integral component of trained immunity. Cell.

[bib31] Murakami M, Kamimura D, Hirano T (2019). Pleiotropy and specificity: insights from the interleukin 6 family of cytokines. Immunity.

[bib32] Netea MG, Quintin J, van der Meer JWM (2011). Trained Immunity: a memory for innate host defense. Cell Host & Microbe.

[bib33] Netea MG, Joosten LAB, Latz E, Mills KHG, Natoli G, Stunnenberg HG, O’Neill LAJ, Xavier RJ (2016). Trained immunity: a program of innate immune memory in health and disease. Science.

[bib34] Netea MG, Schlitzer A, Placek K, Joosten LAB, Schultze JL (2019). Innate and adaptive immune memory: an evolutionary continuum in the host’s response to pathogens. Cell Host & Microbe.

[bib35] Netea MG, Domínguez-Andrés J, Barreiro LB, Chavakis T, Divangahi M, Fuchs E, Joosten LAB, van der Meer JWM, Mhlanga MM, Mulder WJM, Riksen NP, Schlitzer A, Schultze JL, Stabell Benn C, Sun JC, Xavier RJ, Latz E (2020). Defining trained immunity and its role in health and disease. Nature Reviews. immunology.

[bib36] Novakovic B, Habibi E, Wang S-Y, Arts RJW, Davar R, Megchelenbrink W, Kim B, Kuznetsova T, Kox M, Zwaag J, Matarese F, van Heeringen SJ, Janssen-Megens EM, Sharifi N, Wang C, Keramati F, Schoonenberg V, Flicek P, Clarke L, Pickkers P, Heath S, Gut I, Netea MG, Martens JHA, Logie C, Stunnenberg HG (2016). β-glucan reverses the epigenetic state of LPS-induced immunological tolerance. Cell.

[bib37] Ołdakowska M, Ściskalska M, Kepinska M, Marek G, Milnerowicz H (2022). Association of genetic variants in *IL6* Gene (rs1800795) with the concentration of inflammatory markers (IL-6, hs-CRP) and superoxide dismutase in the blood of patients with acute pancreatitis-preliminary findings. Genes.

[bib38] Ostuni R, Piccolo V, Barozzi I, Polletti S, Termanini A, Bonifacio S, Curina A, Prosperini E, Ghisletti S, Natoli G (2013). Latent enhancers activated by stimulation in differentiated cells. Cell.

[bib39] Pancer Z, Cooper MD (2006). The evolution of adaptive immunity. Annual Review of Immunology.

[bib40] Park MD, Le Berichel J, Hamon P, Wilk CM, Belabed M, Yatim N, Saffon A, Boumelha J, Falcomatà C, Tepper A, Hegde S, Mattiuz R, Soong BY, LaMarche NM, Rentzeperis F, Troncoso L, Halasz L, Hennequin C, Chin T, Chen EP, Reid AM, Su M, Cahn AR, Koekkoek LL, Venturini N, Wood-Isenberg S, D’souza D, Chen R, Dawson T, Nie K, Chen Z, Kim-Schulze S, Casanova-Acebes M, Swirski FK, Downward J, Vabret N, Brown BD, Marron TU, Merad M (2024). Hematopoietic aging promotes cancer by fueling IL-1⍺-driven emergency myelopoiesis. Science.

[bib41] Pascutti MF, Erkelens MN, Nolte MA (2016). Impact of viral infections on hematopoiesis: From beneficial to detrimental effects on bone marrow output. Frontiers in Immunology.

[bib42] Patel AA, Ginhoux F, Yona S (2021). Monocytes, macrophages, dendritic cells and neutrophils: an update on lifespan kinetics in health and disease. Immunology.

[bib43] Pellicci DG, Koay HF, Berzins SP (2020). Thymic development of unconventional T cells: how NKT cells, MAIT cells and γδ T cells emerge. Nature Reviews. Immunology.

[bib44] Philips RL, Wang Y, Cheon H, Kanno Y, Gadina M, Sartorelli V, Horvath CM, Darnell JE, Stark GR, O’Shea JJ (2022). The JAK-STAT pathway at 30: much learned, much more to do. Cell.

[bib45] Pietras EM, Mirantes-Barbeito C, Fong S, Loeffler D, Kovtonyuk LV, Zhang S, Lakshminarasimhan R, Chin CP, Techner JM, Will B, Nerlov C, Steidl U, Manz MG, Schroeder T, Passegué E (2016). Chronic interleukin-1 exposure drives haematopoietic stem cells towards precocious myeloid differentiation at the expense of self-renewal. Nature Cell Biology.

[bib46] Quintin J, Saeed S, Martens JHA, Giamarellos-Bourboulis EJ, Ifrim DC, Logie C, Jacobs L, Jansen T, Kullberg B-J, Wijmenga C, Joosten LAB, Xavier RJ, van der Meer JWM, Stunnenberg HG, Netea MG (2012). Candida albicans infection affords protection against reinfection via functional reprogramming of monocytes. Cell Host & Microbe.

[bib47] Schulte-Schrepping J, Reusch N, Paclik D, Baßler K, Schlickeiser S, Zhang B, Krämer B, Krammer T, Brumhard S, Bonaguro L, De Domenico E, Wendisch D, Grasshoff M, Kapellos TS, Beckstette M, Pecht T, Saglam A, Dietrich O, Mei HE, Schulz AR, Conrad C, Kunkel D, Vafadarnejad E, Xu C-J, Horne A, Herbert M, Drews A, Thibeault C, Pfeiffer M, Hippenstiel S, Hocke A, Müller-Redetzky H, Heim K-M, Machleidt F, Uhrig A, Bosquillon de Jarcy L, Jürgens L, Stegemann M, Glösenkamp CR, Volk H-D, Goffinet C, Landthaler M, Wyler E, Georg P, Schneider M, Dang-Heine C, Neuwinger N, Kappert K, Tauber R, Corman V, Raabe J, Kaiser KM, Vinh MT, Rieke G, Meisel C, Ulas T, Becker M, Geffers R, Witzenrath M, Drosten C, Suttorp N, von Kalle C, Kurth F, Händler K, Schultze JL, Aschenbrenner AC, Li Y, Nattermann J, Sawitzki B, Saliba A-E, Sander LE, Deutsche COVID-19 OMICS Initiative (2020). Severe COVID-19 is marked by a dysregulated myeloid cell compartment. Cell.

[bib48] Smith AJP, Humphries SE (2009). Cytokine and cytokine receptor gene polymorphisms and their functionality. Cytokine & Growth Factor Reviews.

[bib49] Sun SJ, Aguirre-Gamboa R, de Bree LCJ, Sanz J, Dumaine A, van der Velden W, Joosten LAB, Khader S, Divangahi M, Netea MG, Barreiro LB (2024). BCG vaccination alters the epigenetic landscape of progenitor cells in human bone marrow to influence innate immune responses. Immunity.

[bib50] Swann JW, Olson OC, Passegué E (2024). Made to order: emergency myelopoiesis and demand-adapted innate immune cell production. Nature Reviews. Immunology.

[bib51] Vierbuchen T, Ling E, Cowley CJ, Couch CH, Wang X, Harmin DA, Roberts CWM, Greenberg ME (2017). AP-1 transcription factors and the BAF complex mediate signal-dependent enhancer selection. Molecular Cell.

[bib52] Vuscan P, Kischkel B, Joosten LAB, Netea MG (2024). Trained immunity: general and emerging concepts. Immunological Reviews.

[bib53] Yamanaka R, Barlow C, Lekstrom-Himes J, Castilla LH, Liu PP, Eckhaus M, Decker T, Wynshaw-Boris A, Xanthopoulos KG (1997). Impaired granulopoiesis, myelodysplasia, and early lethality in CCAAT/enhancer binding protein ɛ-deficient mice. PNAS.

[bib54] Yamashita M, Passegué E (2019). TNF-α coordinates hematopoietic stem cell survival and myeloid regeneration. Cell Stem Cell.

[bib55] Zeng T, Spence JP, Mostafavi H, Pritchard JK (2024). Bayesian estimation of gene constraint from an evolutionary model with gene features. Nature Genetics.

[bib56] Zhang H, Nguyen-Jackson H, Panopoulos AD, Li HS, Murray PJ, Watowich SS (2010). STAT3 controls myeloid progenitor growth during emergency granulopoiesis. Blood.

[bib57] Zhao JL, Ma C, O’Connell RM, Mehta A, DiLoreto R, Heath JR, Baltimore D (2014). Conversion of danger signals into cytokine signals by hematopoietic stem and progenitor cells for regulation of stress-induced hematopoiesis. Cell Stem Cell.

[bib58] Zhao Y, Vartak SV, Conte A, Wang X, Garcia DA, Stevens E, Kyoung Jung S, Kieffer-Kwon KR, Vian L, Stodola T, Moris F, Chopp L, Preite S, Schwartzberg PL, Kulinski JM, Olivera A, Harly C, Bhandoola A, Heuston EF, Bodine DM, Urrutia R, Upadhyaya A, Weirauch MT, Hager G, Casellas R (2022). “Stripe” transcription factors provide accessibility to co-binding partners in mammalian genomes. Molecular Cell.

